# Supplementary material for: Controlling pairing of π-conjugated electrons in 2D covalent organic radical frameworks via in-plane strain
Source: Nat Commun. 2021 Mar 17;12:1705. doi: 10.1038/s41467-021-21885-y (PMC7969611; doi:10.1038/s41467-021-21885-y)
Supplement: Supplementary file 1 — Supplementary Information [file 41467_2021_21885_MOESM1_ESM.pdf]

Supplementary Information

Controlling Pairing of π-Conjugated Electrons in 2D Covalent Organic Radical Frameworks via In-plane Strain

Isaac Alcón[1,*], Raúl Santiago[2], Jordi Ribas-Arino[2], Mercè Deumal[2], Iberio de P.R. Moreira[2] and Stefan T. Bromley[2,3,*]

[1]*Institut für Chemie und Biochemie, Physikalische und Theoretische Chemie, Freie Universität Berlin, Arnimallee 22, 14195 Berlin, Germany*

[2]*Departament de Ciència de Materials i Química Física & Institut de Química Teòrica i Computacional (IQTCUB), Universitat de Barcelona, c/ Martí i Franquès 1-11, 08028 Barcelona, Spain*

[3]*Institució Catalana de Recerca i Estudis Avançats (ICREA), Passeig Lluís Companys 23, 08010 Barcelona, Spain*

* Corresponding authors: ialcon8@gmail.com, s.bromley@ub.edu

Section 1. Results at 0K

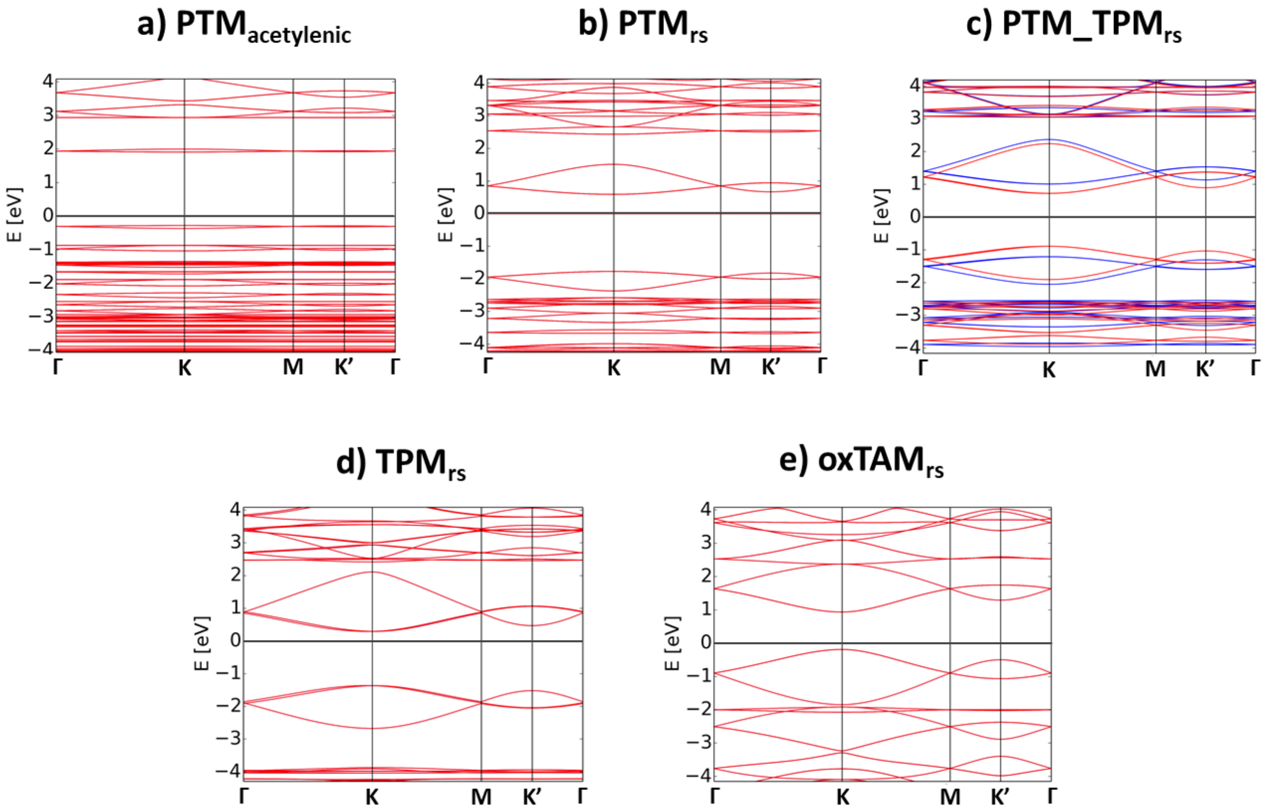

Fig. S1. Spin-resolved (spin-up: blue; spin-down: red) band structures for the (AFM) open-shell electronic solution of the 2D-CORFs considered in this work. Note that in band structures **a)**, **b)**, **d)** and **e)** spin-up and spin-down bands are exactly superimposed. For **c)**, due to the different chemical functionalization of *A* (chlorinated aryl rings) and *B* (hydrogenated aryl rings) lattices, the AFM spin degeneracy is lifted. The Fermi level is indicated with a horizontal black line.

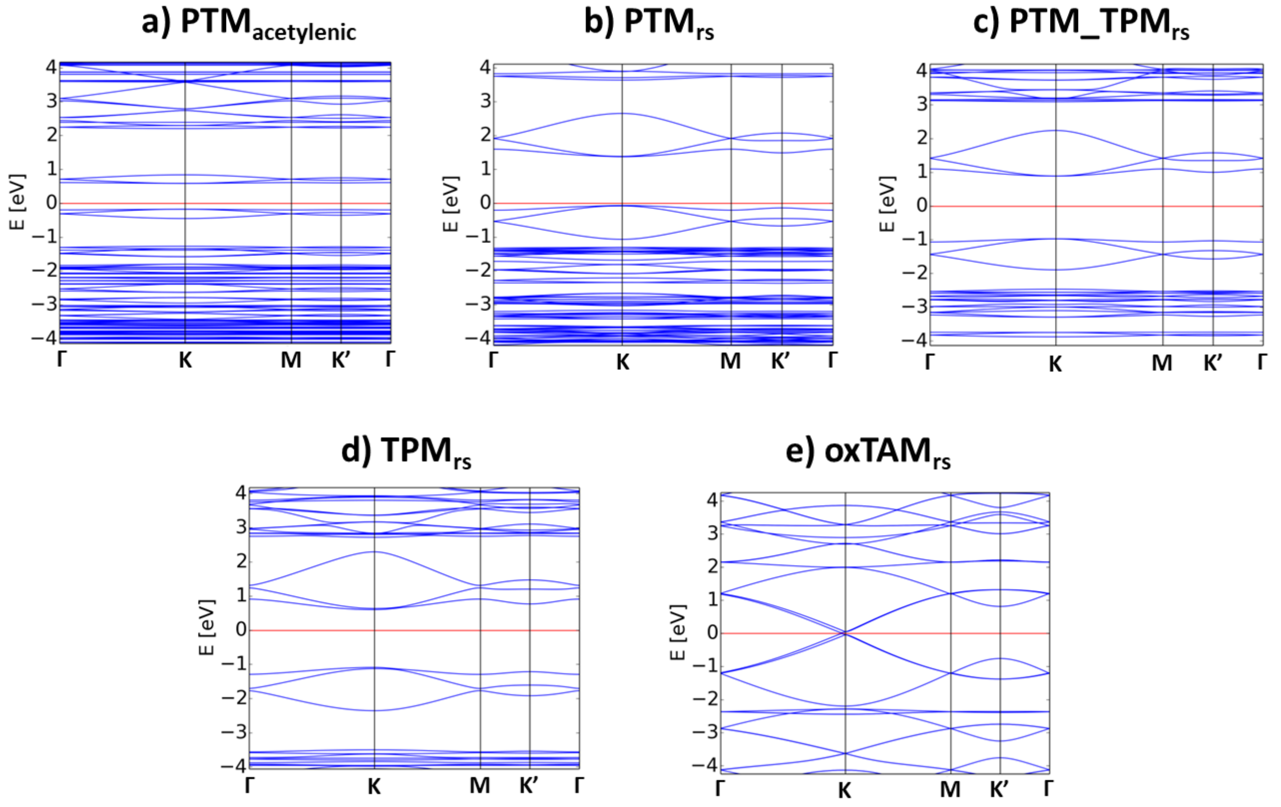

Fig. S2. Band structures for the closed-shell (quinoidal) electronic solution of the 2D-CORFs considered in this work. The presence of a semimetallic Dirac cone for oxTAM$_{rs}$ (**e**) reveals its tendency to favour delocalization as opposed to local electron pairing. The Fermi level is indicated with a horizontal red line.

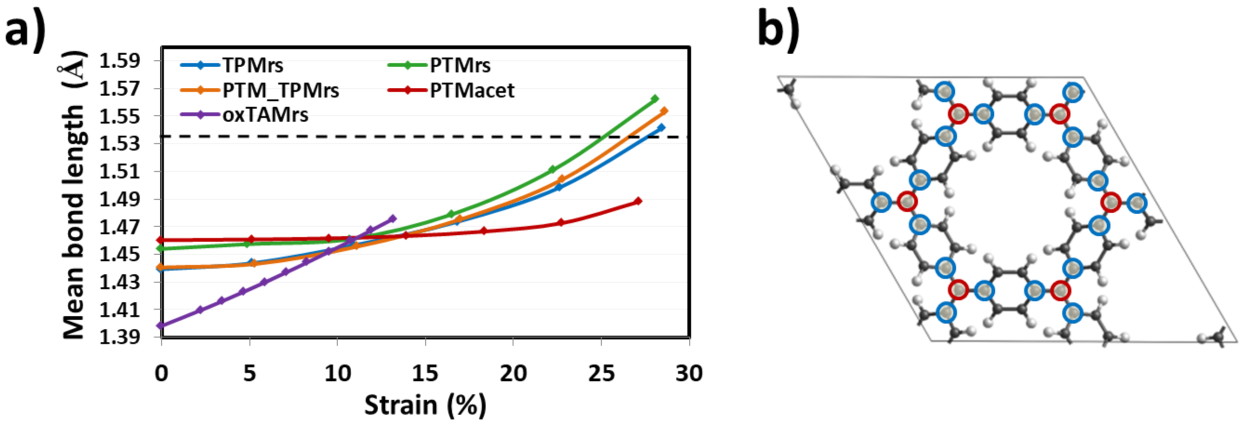

Fig. S3. **a)** Mean bond length (Å) between αC atoms (red circle in **b**) and their first nearest neighbours (blue circle in **b**) against uniaxial strain for all considered 2D-CORFs. The dotted horizontal line is the C-C distance in ethane (1.535Å),[1] as a prototypical length for the single C-C bond.

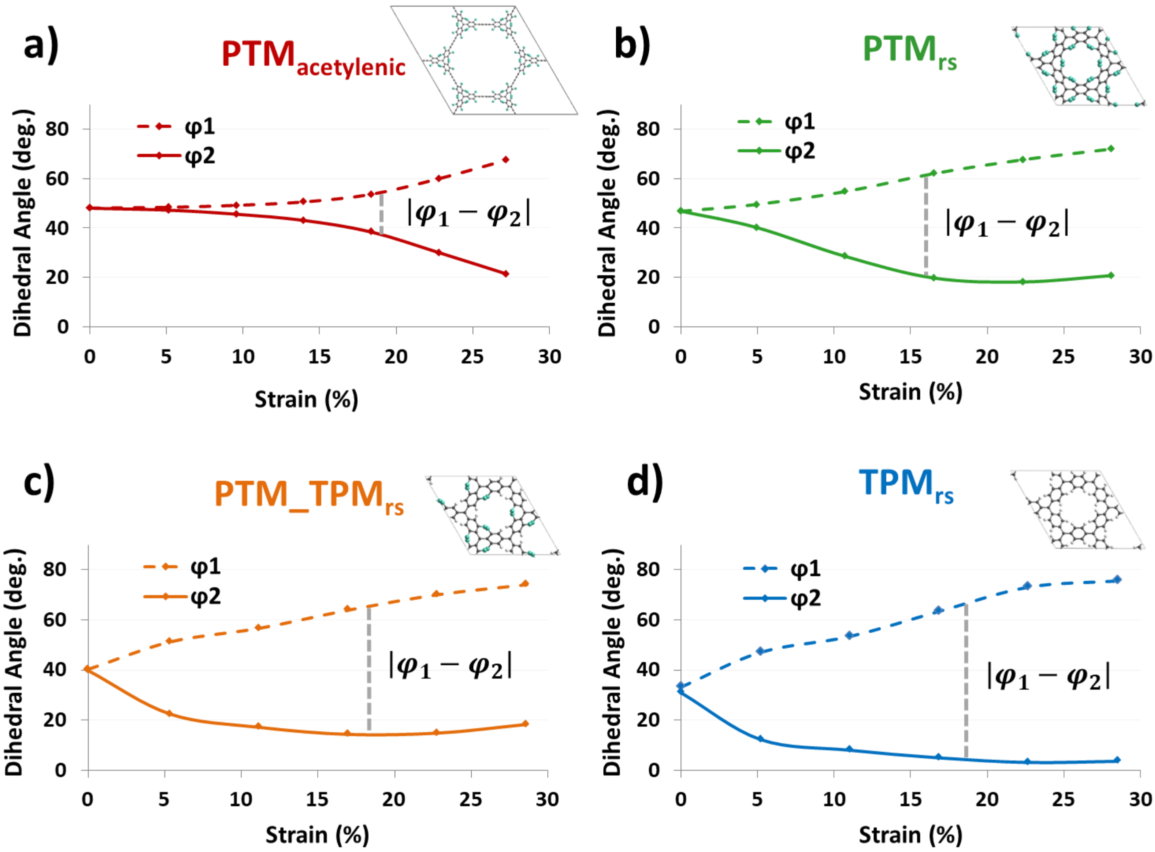

Fig. S4. Dihedral angle (degrees) *vs*. uniaxial strain (%) for the aryl rings parallel (φ_2) and non-parallel (φ_1) to the strain direction. The difference between the two angles ($|\varphi_1 - \varphi_2|$; vertical dashed grey lines) *vs*. strain is presented in **Fig. 3** in the main text.

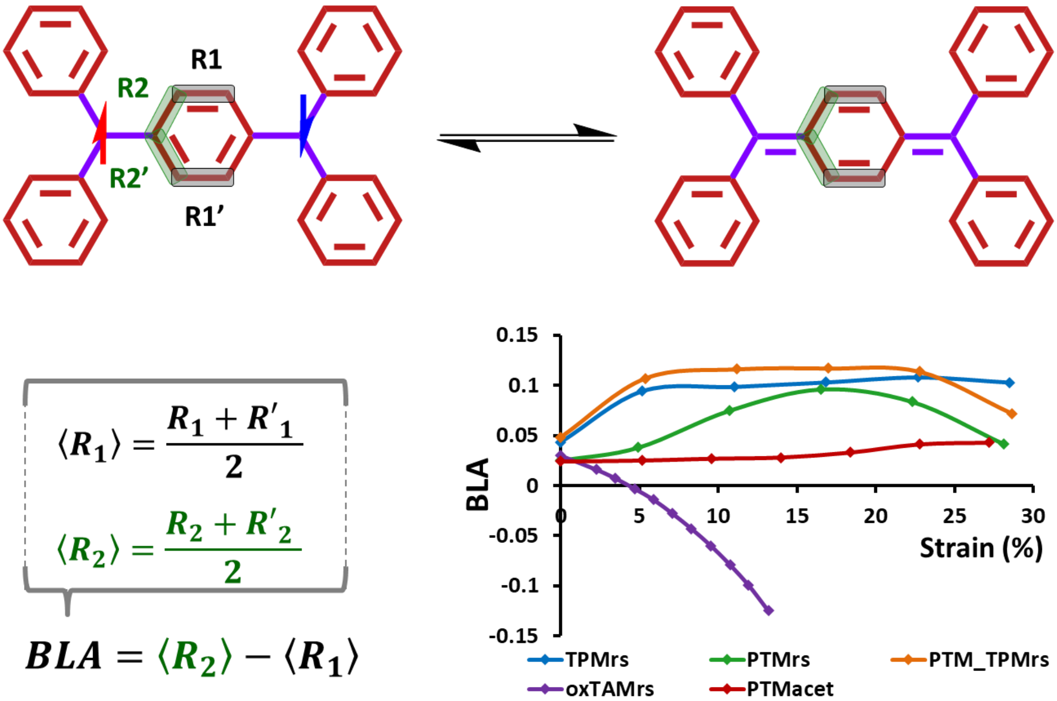

Fig. S5. Bond length alternation (BLA) vs. uniaxial strain calculated from the difference between bond lengths of type R_1 and R_2 within aryl rings accommodating electron pairing (i.e. those parallel to the strain direction).

2D Material	Calculated in-plane Young's Modulus (GPa)	% relative to graphene	Approx. multiplicative factor relative to graphene
**$PTM_{acetylenic}$**	<u>2.1</u>	0.2	$\frac{1}{458}$
**TPM_{rs}**	<u>31.4</u>	3.3	$\frac{1}{31}$
**PTM_TPM_{rs}**	<u>33.4</u>	3.5	$\frac{1}{29}$
**PTM_{rs}**	<u>46.7</u>	4.9	$\frac{1}{21}$
**MoS_2**	*265±13(exp)*[2]	27.5±1	$\frac{1}{3.6}$
**$oxTAM_{rs}$**	<u>651.8</u>	67.7	$\frac{1}{1.5}$
**graphene**	<u>962.6</u>, *1024(theory)*[3], *1020±150(exp.)*[4]	100	1

Table S1. Comparison of our calculated in-plane Young's Modulus (YM) values for our considered 2D-CORF materials as compared to that of graphene and single layer MoS_2. Our calculated YM values are underlined and YM values from other recent experimental and theoretical studies values (for MoS_2 and graphene) are in italics. For our 2D-CORFs we use a strain range of 0-25% and the supercells reported in the main text. The thickness of each 2D-CORF material is taken to be the sum of the maximal interatomic distance in the out-of-plane direction and twice the van der Waals radius of the outermost atoms. For graphene we use a smaller 0 - 8% range of strain, with an 18 atom (3×3×1) supercell, and take the thickness to be the interlayer separation distance in graphite. In our calculations we use our computed total energy versus strain data which can be well approximated as being in the elastic and linear regime (i.e. Hooke's Law response to strain). Under this approximation we used the relation: $E(\Delta L) = \frac{k}{2}\Delta L^2$ – where ΔL is the elongation with respect to strain (in the stretching direction), $E(\Delta L)$ is the resultant change in total energy with respect to the strain-induced elongation, and k is the spring constant. A quadratic fit to our calculated $E(\Delta L)$ versus ΔL data gives us a value of k, from which we derive the YM values via: $\mathrm{YM} = \frac{kL_0}{A}$, where L_0 is the initial relaxed length of the material and A is the cross sectional area.

2D-CORF	$E_{AFM} - E_{FM}$ (meV/αC)	Magnetic coupling, *J* (meV)
**$PTM_{acetylenic}$**	-25	-17 *(-15)*
**PTM_{rs}**	-213	-142 *(-157)*
**PTM_TPM_{rs}**	-353	-235
**TPM_{rs}**	-476	-317 *(-318)*
**$oxTAM_{rs}$**	-555	-370

Table S2. Calculated magnetic couplings (*J*) for all considered 2D-CORFs. The Heisenberg Hamiltonian is given by: $\hat{H} = -2J\sum_{i>j}\hat{S}_i \cdot \hat{S}_j$ where the sum runs over all different pairs of spins of directly connected centres. If the strong localization limit of the mapping is taken, it is assumed that the diagonal form of the Heisenberg is valid (i.e. the Ising model in which only the $\hat{S}_i^z$ components of the spin operators appear), and thus: $\hat{H} = -2J\sum_{i>j}\hat{S}_i^z\hat{S}_j^z$. From this 2D model it follows that: $E_{AF} - E_{FM} = 2zS^2J$, where *z* is the number

of nearest neighbours of a given centre connected by *J* and the energies are given per magnetic centre. In the case of our 2D-CORFs, we take an array of S = ½ magnetic centres (i.e. radical αC centres) on the 2D honeycomb lattice where z = 3, we then have: $J = \frac{E_{AF}-E_{FM}}{2zS^2} = \frac{2}{3}(E_{AF} - E_{FM})$. In the table we include calculated $E_{AF} - E_{FM}$ values and the corresponding values of *J* for all considered relaxed (i.e. unstrained) 2D-CORFs. In parentheses we also include values obtained for three 2D-CORFs reported in ref. 8. We note that the present *J* values are based on supercells using six αC centres, rather than supercells using two αC centres in ref. 8. As larger supercells provide more degrees of freedom for structural relaxation, the present J values should be taken as being slightly more refined results.

Section 2. Results at 300K

Fig. S6 presents the dihedral angle variations associated with each aryl ring within the unit cell (nine in total) throughout the 3ps AIMDS runs for TPM_{rs}, PTM_TPM_{rs} and PTM_{rs} at different strains (ε = 0%, 16% and 28%). Overall, all 2D-CORFs present the same global behavior. In the relaxed conformation (ε = 0%) we see that TPM_{rs} (**S6a**) exhibits significantly higher dihedral angle fluctuations than the other two 2D-CORFs. This difference is due to the smaller steric hindrance of phenyl rings (H-functionalized) compared to perchlorinated rings (Cl-functionalized), leading to a higher conformational flexibility within TPM_{rs}. Such differences were also found for the associated triarylmethyl molecules[5] (the triphenylmethyl[6] and perchlorotriarylmethyl,[7] respectively), which points to the generality of this effect. Upon stretching we observe the same behavior as found at 0K (**Fig. 3** in the main text): i.e. aryl rings bridging αC centers along the stretching direction become more planar with strain (see 2 in the structures at the top of **Fig. S6a**) whereas the non-parallel ones get twisted towards higher out-of-plane dihedral angles (see 1 in the structures at the top of **Fig. S6a**). Indeed, for both ε = 17% and 28 % we see there is a dihedral angle splitting for the three 2D-CORFs. For TPM_{rs}, the flattened aryl rings (2) are stabilized into a nearly flat conformation (i.e. dihedral angles fluctuate very close to 0°), whereas the other set of aryl rings become highly perpendicular, with dihedral angles close to 80° (**Fig. S6c**).

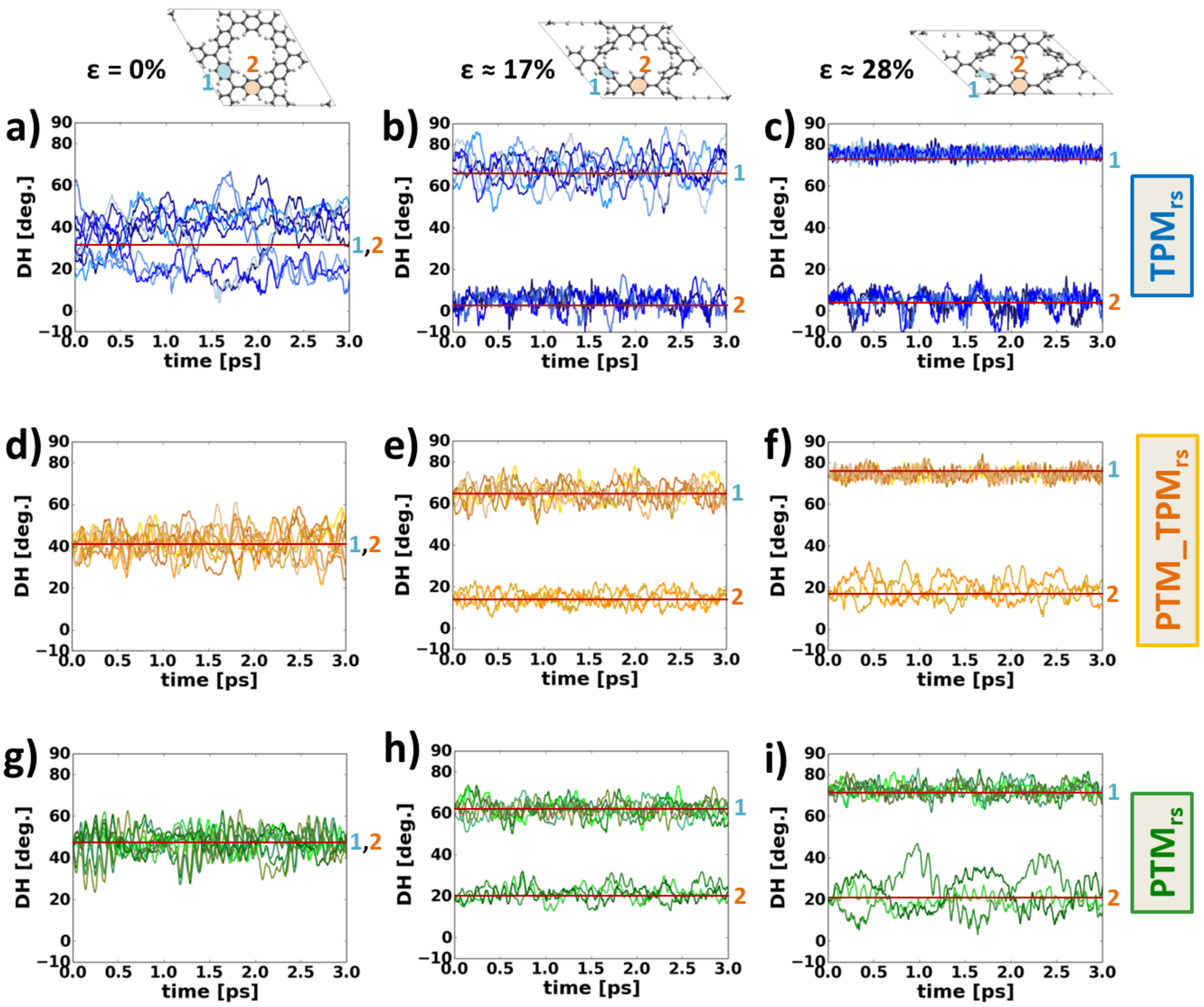

Fig. S6. Time-resolved evolution of dihedral angles (DH) of each of the nine aryl rings within the unit cell during 3ps AIMDS at 300K, at different uniaxial strains (columns) for the **(a, b, c)** TPM_{rs}, **(d, e, f)** PTM_TPM_{rs} and **(g, h, i)** PTM_{rs}. Horizontal red lines indicate the averaged values of aryl ring types 1 and 2 (see inset structures at the top) for the corresponding optimized structures at 0K.

Such general behavior is also found for the PTM_TPM_{rs} and PTM_{rs}, but with lower torsional differences due to the lower conformational flexibility caused by steric hindrance effects, as explained above. Finally, another general trend we observe is the larger rotations for flattened aryl rings for the highest applied strains (ε = 28%) compared with the remaining aryl rings (see **Fig. S6c, f, i**). Once more, the explanation for this effect is steric hindrance. In these highly stretched conformations the two out-of-plane aryl rings are very close to each other, increasing steric hindrance and significantly decreasing their fluctuation freedom. Conversely, flattened aryl rings become further away from the other "out-of-plane" rings with increasing strain, which gives them a higher rotational flexibility (lower steric hindrance). In such "sterically-free" situation, we may see that aryl rings in PTM_{rs} show more significant fluctuations than for the TPM_{rs}, which cannot be explained by steric-hindrance effects. We believe this apparent contradiction shows the effect of the significant weight differences between H atoms and Cl atoms. Cl atoms have a much higher inertia (higher atomic mass) and so their thermal vibrations lead to higher rotational motion, as compared with the lighter (and so less perturbative) H atoms. Overall, results of **Fig. S6** demonstrate that the external manipulation of aryl ring dihedral angles via uniaxial strain operates efficiently under the effect of thermal fluctuations at room temperature (300K).

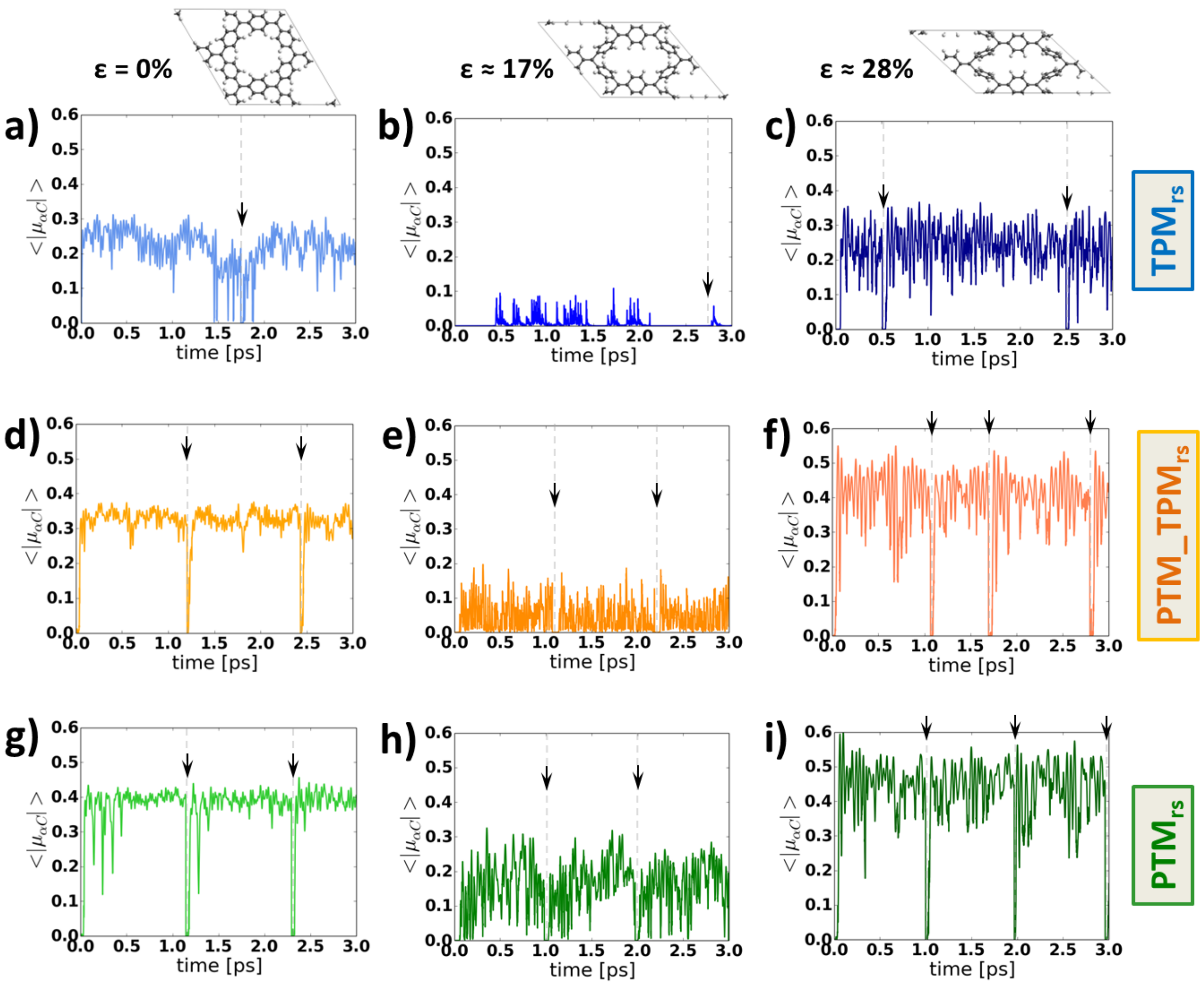

Fig. S7. Time-resolved evolution of αC averaged absolute spin population during 3ps AIMDS at 300K at different uniaxial strains for the **(a, b, c)** TPM_{rs}, **(d, e, f)** PTM_TPM_{rs} and **(g, h, i)** PTM_{rs}. The arrows and dashed lines indicate re-starting points during each AIMDS, causing a sudden (and unphysical) decrease of absolute spin due to the non-spin polarized initial guess used in these calculations.

We note that the vertical dashed lines in **Fig. S7** indicate AIMD re-starting points, which lead to sudden artificial depletions of $\langle|\mu_{\alpha C}|\rangle$. We have removed the data around such re-start times (±0.03ps) in the results presented in the main text (**Fig. 5**) to provide a clearer visualization of the physical response of the systems at finite temperatures. **Fig. S7** indicates such restart times (vertical dashed lines) plotting the raw data.

Section 3. On the on the performance of PBE0 in describing magnetic coupling in a benchmark molecular system.

In order to justify the selected hybrid DFT approach used in this work, we have analysed the electronic structure and magnetic coupling in a molecular system synthesised by Li et al.[8] for which both experimental X-ray structure and magnetic properties (from susceptibility and EPR studies) have been reported. This "superbenzene" molecular system has a ring structure of six αC centres connected via shared phenyl groups as found in TPM_{rs}. These authors reported an energy difference between the lowest singlet and triplet states of this molecule of a ΔE_{S-T} = −6.23 kcal/mol = -270 meV estimated from the temperature dependence of the intensity of EPR peaks fitted to a Bleaney-Bowers equation for a spin dimer model. Although this assumption can be questionable, the fact that the obtained energy is significantly large

justifies the simplification of the spectrum of magnetic states of this magnetic system formed by a ring of six S=1/2 coupled spins to the lowest singlet and triplet states to analyse the magnetic response of the system (i.e. the remaining singlet, triplet, quintet, and septet excited states are not thermally accessible and, hence, ignored). To relate this estimated $\Delta E_{S\text{-}T}$ energy difference with the correct Heisenberg model of the system we have to map this energy to the exact solution for an AF hexagonal Heisenberg system of S=1/2 spin particles interacting via $-2JS_iS_j$ nearest neighbour operators.[9] The exact solution for this spin model gives $\Delta E_{S\text{-}T}$ = 1.369J leading to J = -197 meV. Our calculations using unrestricted PBE0 DFT calculations within the broken-symmetry approach and employing the experimental structure provide a value of J = -249 meV. We note that the agreement with experiment is rather good considering the small energy scales involved and the uncertainty in determining accurate experimental $\Delta E_{S\text{-}T}$ values.

We can further compare this J value for the "superbenzene" molecular system for that obtained for TPM_{rs}, since it represents the magnetic coupling between two nearest neighbour αC spin centres with the same linkers and local environment. In order to do, we first need to account for the difference in the dihedral angles of the aryl rings in each system. As described previously by some of us,[10] average dihedral angles of aryl rings in play an important role in determining J values in this type of system. The average dihedral angle for unstrained TPM_{rs} is around 32-33 degrees compared to (69.1 + 2*26.6)/3 = 40.8 degrees for the experimental structure of the molecular system. Taking into account the 7-8 degrees difference in the average dihedral angle,[11] an estimated J value of -240 meV is obtained for TPM_{rs}. Note that J = -317 meV is obtained for fully relaxed TPM_{rs} with relatively flatter aryl rings (see **table S2**). The good correspondence between the J values calculated by means of our DFT calculations using the PBE0 functional for TPM_{rs} and the corresponding experimental and calculated values of the "superbenzene" molecular system provides a strong argument to support the use of the PBE0 functional to describe the electronic structure and magnetic properties of this type of system.

Section 4. Optimized geometries and single-point input files (FHI-AIMS format)

<u>Optimized geometry for the TPM_{rs} 2D-CORF</u>

```
lattice_vector    17.21330103    -0.05726972     0.00000000
lattice_vector    -8.64715339    14.86898460     0.00000000
lattice_vector     0.00000000     0.00000000    40.00000000
atom     9.94428624     0.26819511    18.79447990 C
atom    10.86910259     0.19542750    18.23665197 H
atom     0.80391315    13.99070804    19.45410117 C
atom    -0.43950503    14.11401107    20.11149636 C
atom     7.50974022     0.42603222    20.10982024 C
atom    -0.82325741    13.26920711    20.66894183 H
atom     6.58491784     0.49885890    20.66763555 H
atom     9.24639410     1.44920212    18.79283953 C
atom     9.63027667     2.29398359    18.23545542 H
atom     7.25133759    12.70886477    19.45263293 C
atom     5.81601446    12.71593127    19.45293860 C
atom     7.97471102    13.95742660    19.45201937 C
atom     5.06823199    11.68834096    18.83433235 C
atom     5.08337887    13.75791469    20.06692853 C
atom     3.71379768    13.77098299    20.06404276 C
```

atom	2.96599007	12.74178743	19.44800753	C
atom	3.69870111	11.70149917	18.83135505	C
atom	5.59370115	10.90438077	18.30436328	H
atom	3.16111193	10.92698900	18.29981800	H
atom	3.18852841	14.55625291	20.59231394	H
atom	5.62108894	14.53367393	20.59657312	H
atom	10.85939682	10.09795519	18.26559332	H
atom	9.62111664	12.19611651	18.26399747	H
atom	6.54743215	10.38548533	20.64976962	H
atom	7.78588906	8.28739889	20.65150844	H
atom	7.79838948	7.23532717	18.23131542	H
atom	6.57648033	5.12569640	18.22796261	H
atom	9.62812071	3.35312752	20.66812320	H
atom	10.85005322	5.46306892	20.67159955	H
atom	11.78066530	5.96501624	18.28158816	H
atom	7.47760209	10.31560038	20.10063638	C
atom	7.97800428	11.46652807	19.45331430	C
atom	9.23045853	11.34769058	18.81095692	C
atom	9.92800087	10.16712272	18.81282965	C
atom	8.17517841	9.13515480	20.10258997	C
atom	9.23923462	4.19554405	20.11071819	C
atom	7.99962016	4.06441065	19.44962479	C
atom	7.49944632	5.20612461	18.78776534	C
atom	8.18723071	6.39312402	18.78921164	C
atom	9.92693395	5.38242698	20.11220853	C
atom	12.31860336	6.73297118	18.82232621	C
atom	9.42589398	9.01532477	19.45736296	C
atom	9.42632595	6.52447441	19.45164481	C
atom	11.58672536	7.76501387	19.45172664	C
atom	10.14975444	7.77139312	19.45342004	C
atom	1.53054970	12.74641346	19.45286017	C
atom	8.00313928	1.57260621	19.45047659	C
atom	7.27661944	2.81698240	19.45204983	C
atom	-1.34073008	7.79067978	19.45503823	C
atom	-2.77770740	7.79683623	19.45712103	C
atom	-0.61771637	9.03806031	19.45587581	C
atom	-3.52454010	6.77739241	18.82543047	C
atom	-3.50963954	8.82887764	20.08676266	C
atom	-4.87976540	8.84172478	20.08368399	C
atom	0.62130617	9.17033464	20.11811681	C
atom	-1.11898182	10.17944327	18.79473283	C
atom	-0.43185834	11.36669603	18.79540873	C
atom	0.80778833	11.49878110	19.45622367	C
atom	1.30846000	10.35772015	20.11885112	C
atom	-2.99884159	5.99988095	18.28624360	H
atom	-5.40545697	9.61928205	20.62280692	H
atom	-2.97175035	9.59695091	20.62738646	H
atom	1.01061225	8.32860510	20.67640691	H
atom	2.23139002	10.43892909	20.67858877	H
atom	-0.82123896	12.20858157	18.23754028	H
atom	-2.04220047	10.09805692	18.23560486	H

atom 2.26031179 5.17589398 18.25716436 H
atom 1.02285558 7.27465262 18.25659176 H
atom -2.05060202 5.46461182 20.64301816 H
atom -0.81345041 3.36577274 20.64330124 H
atom -0.77813605 2.30709486 18.20872082 H
atom 2.24122545 0.55494547 20.70178031 H
atom 3.18630799 1.02889427 18.30864085 H
atom 5.61897578 1.00753350 18.31162907 H
atom 5.64590684 4.63542358 20.60629208 H
atom 3.21321345 4.65691917 20.60284270 H
atom -1.11947550 5.39513484 20.09537027 C
atom -0.61699844 6.54694745 19.45101838 C
atom 0.63346553 6.42686438 18.80537965 C
atom 1.33037908 5.24608406 18.80664095 C
atom -0.42260347 4.21422658 20.09651755 C
atom -0.39671595 1.46846715 18.77702850 C
atom 1.32525299 0.46909150 20.13140178 C
atom 5.10836332 3.86104170 20.07452573 C
atom 5.84112748 2.82142523 19.45695169 C
atom 5.09356799 1.79223945 18.84059218 C
atom 3.72397219 1.80461031 18.83840052 C
atom 3.73882773 3.87352165 20.07220143 C
atom 0.82959092 4.09515489 19.45371694 C
atom 0.83262257 1.60420666 19.45460347 C
atom 2.99130512 2.84595457 19.45329688 C
atom 1.55595559 2.85274731 19.45399163 C
atom 15.21338424 0.13767748 18.20466189 H
atom 16.12928725 0.22353408 18.77514718 C
atom -7.62788833 13.31186537 20.69800700 H
atom -8.00928327 14.15045913 20.12963784 C

Optimized geometry for the PTM_{rs} 2D-CORF

lattice_vector 17.30296467 0.00039308 0.00000000
lattice_vector -8.66258790 14.98973977 0.00000000
lattice_vector 0.00000000 0.00000000 40.00000000
atom -7.89532545 14.54197334 20.93998295 C
atom -7.50920288 13.37387701 22.14681639 Cl
atom 16.64665112 0.37193061 19.18877454 C
atom 15.44433214 0.11817232 17.98047961 Cl
atom 1.83912085 3.06178873 20.06484437 C
atom 3.29304127 3.06186953 20.06391504 C
atom 1.11313473 1.80175916 20.06536873 C
atom 1.11193157 4.32046643 20.06487295 C
atom 4.03185780 3.88327811 20.93903837 C
atom 4.03099777 2.23961194 19.18884863 C
atom 5.41498723 2.23815152 19.18944957 C
atom 6.15381830 3.05904700 20.06501442 C
atom 5.41584412 3.88144780 20.93996137 C
atom 1.45681011 0.75224635 20.94086889 C

atom	0.03326603	1.57158475	19.18957040	C
atom	0.03049047	4.54823743	20.93978412	C
atom	1.45440936	5.37143708	19.19040453	C
atom	0.76206227	6.56971215	19.19083355	C
atom	-0.31903380	6.79764820	20.06602712	C
atom	-0.66152914	5.74670918	20.94054132	C
atom	3.21287083	4.80287652	22.14476084	Cl
atom	6.23569285	4.79714772	22.14810621	Cl
atom	6.23404850	1.32001018	17.98272695	Cl
atom	3.21108444	1.32406282	17.98077087	Cl
atom	2.65897881	1.00596127	22.14933822	Cl
atom	-0.35328759	2.73969858	17.98288969	Cl
atom	-0.35435104	3.37911018	22.14601541	Cl
atom	-1.86628197	5.99680464	22.14726874	Cl
atom	1.14597007	7.73825212	17.98374669	Cl
atom	2.65920243	5.12140962	17.98373115	Cl
atom	-1.86837036	10.11392232	17.98311182	Cl
atom	-0.35927436	12.73374847	17.98352738	Cl
atom	2.65481872	10.99668943	22.14764151	Cl
atom	1.14536802	8.37774767	22.14760061	Cl
atom	-2.41990662	9.79385540	22.14898203	Cl
atom	-5.44271609	9.79742778	22.14548847	Cl
atom	-2.41879191	6.31524708	17.98487384	Cl
atom	1.45075660	10.74489975	20.94052367	C
atom	1.10722977	11.79490577	20.06550975	C
atom	0.02657267	11.56545625	19.19032018	C
atom	-0.66425408	10.36613938	19.18997535	C
atom	0.75971550	9.54577339	20.94038246	C
atom	-4.62304774	8.87891945	20.93950428	C
atom	-3.23906243	8.87769299	20.94084112	C
atom	-3.23855445	7.23383361	19.19074780	C
atom	-0.32086425	9.31624648	20.06513795	C
atom	-2.50051654	8.05550917	20.06623672	C
atom	-1.04661077	8.05615023	20.06623610	C
atom	7.60778356	3.05876527	20.06454559	C
atom	8.33544271	1.79999529	20.06425361	C
atom	1.83350001	13.05474704	20.06501907	C
atom	10.48799052	8.05711986	20.06396915	C
atom	11.94183675	8.05758229	20.06402034	C
atom	9.76183562	6.79743353	20.06420063	C
atom	9.76044226	9.31589284	20.06382435	C
atom	12.68043374	7.23529995	19.18951251	C
atom	10.10549213	5.74749808	20.93945073	C
atom	8.68131452	6.56772040	19.18907112	C
atom	7.99075283	5.36832380	19.18911197	C
atom	8.33427586	4.31846966	20.06442872	C
atom	9.41480504	4.54812586	20.93952616	C
atom	8.67943464	9.54402835	20.93900043	C
atom	10.10276248	10.36660807	19.18893937	C
atom	9.41070970	11.56514633	19.18943340	C
atom	8.32967100	11.79323789	20.06454550	C

atom 7.98729462 10.74246789 20.93930048 C
atom 11.86137380 6.31891604 17.98148434 Cl
atom 11.30971849 5.99966236 22.14630269 Cl
atom 9.80060824 3.37993803 22.14650753 Cl
atom 6.78694158 5.11594620 17.98185781 Cl
atom 8.29532842 7.73552987 17.98186361 Cl
atom 8.29502259 8.37550627 22.14599937 Cl
atom 6.78289653 10.99305527 22.14618179 Cl
atom 9.79517471 12.73423182 17.98306491 Cl
atom 11.30704404 10.11603470 17.98187787 Cl
atom 6.23029052 14.78781090 22.15002700 Cl
atom 3.20703174 14.79216193 22.14780172 Cl
atom 3.20566673 11.31715325 17.98073559 Cl
atom 6.22877138 11.31259084 17.98358486 Cl
atom 4.02542488 12.23250846 19.18914826 C
atom 3.28747802 13.05443548 20.06449384 C
atom 4.02623993 13.87489456 20.94051102 C
atom 5.41022837 13.87326884 20.94126964 C
atom 5.40942452 12.23086762 19.18998528 C
atom 8.32783259 14.31211717 20.06413101 C
atom 6.14813965 13.05167529 20.06565753 C
atom 7.60214688 13.05190621 20.06489409 C
atom 9.80158999 2.74141179 17.98321894 Cl
atom 9.41734727 1.57258186 19.19001010 C
atom 6.78750141 0.99845319 22.14473872 Cl
atom -0.36004421 13.37243120 22.14647122 Cl
atom 7.99327361 0.74902990 20.93896972 C
atom 0.02399819 14.54098317 20.93940305 C
atom 1.10599377 14.31356359 20.06511332 C
atom 11.31698790 0.12577611 17.98506608 Cl
atom 10.11087508 0.37483147 19.19058482 C

Optimized geometry for the PTM_TPM$_{rs}$ 2D-CORF

lattice_vector 17.23251028 -0.02217514 0.00000000
lattice_vector -8.65130002 14.89229868 0.00000000
lattice_vector 0.00000000 0.00000000 40.00000000
atom 10.13267934 0.43020129 19.28003565 C
atom 11.41378494 0.29865272 18.11351447 Cl
atom 1.09441483 14.20886612 20.06849249 C
atom -0.06729226 14.41629609 20.85555613 C
atom 7.85665830 0.68576974 20.82346682 C
atom -0.57789414 13.23768367 22.02627036 Cl
atom 6.98572838 0.78405397 21.45574667 H
atom 9.47292820 1.63138647 19.30887207 C
atom 9.81422794 2.43863070 18.67654615 H
atom 7.55559150 12.94419173 20.06524545 C
atom 6.11939509 12.94620594 20.06603161 C
atom 8.27406612 14.19128664 20.06450010 C
atom 5.39637121 12.00730030 19.31489717 C

atom	5.40241258	13.89200383	20.81473545	C
atom	4.03253422	13.85952661	20.84400588	C
atom	3.26288576	12.95908580	20.06245259	C
atom	4.02675567	12.05263880	19.28202036	C
atom	5.91834123	11.29853641	18.68783428	H
atom	3.26584424	11.01049159	18.11771041	Cl
atom	11.92869582	0.01858136	22.00345141	Cl
atom	5.92964654	14.59712220	21.44148689	H
atom	-5.83320746	10.24817406	18.12331949	Cl
atom	9.80057852	12.36558973	18.68743503	H
atom	6.95563710	10.70363899	21.44569305	H
atom	8.03816554	8.26421007	22.01604699	Cl
atom	8.05978115	7.69633087	18.09386265	Cl
atom	6.98482618	5.24857292	18.66104054	H
atom	9.80298856	3.62187922	21.46597116	H
atom	11.38578142	5.77641025	22.03252716	Cl
atom	11.88925141	6.05074793	18.10637430	Cl
atom	7.82962541	10.60691809	20.81741939	C
atom	8.27848256	11.70276776	20.06500801	C
atom	9.45529407	11.55631791	19.31502621	C
atom	10.11351196	10.35444405	19.28509691	C
atom	8.55580300	9.44465414	20.85012739	C
atom	9.46175936	4.42542680	20.82889027	C
atom	8.29686648	4.26805397	20.06431275	C
atom	7.85113976	5.35480106	19.29832756	C
atom	8.57046579	6.52197015	19.26853141	C
atom	10.11344103	5.63148820	20.85827805	C
atom	12.65193983	7.08629975	19.27522504	C
atom	9.72130780	9.23787059	20.06776505	C
atom	9.72396984	6.73873851	20.06349812	C
atom	11.88930888	7.98757835	20.06174701	C
atom	10.44606964	7.99037813	20.06427615	C
atom	1.82044442	12.96136961	20.06478082	C
atom	8.30078715	1.77933989	20.06536176	C
atom	7.57810251	3.02161976	20.06640948	C
atom	-1.05050589	7.99443716	20.06221367	C
atom	-2.48747570	7.99689054	20.06389916	C
atom	-0.33156558	9.24092292	20.06290497	C
atom	-3.21069493	7.06346168	19.30645252	C
atom	-3.20365099	8.93681794	20.82012228	C
atom	-4.57376223	8.90441921	20.84915044	C
atom	0.83191984	9.39778451	20.82947497	C
atom	-0.77663562	10.32842929	19.29751418	C
atom	-0.05653311	11.49513915	19.26824604	C
atom	1.09710663	11.71048764	20.06347296	C
atom	1.48439034	10.60344911	20.85929193	C
atom	-2.68858446	6.35944023	18.67419049	H
atom	-5.32964830	9.94846753	22.01462295	Cl
atom	-2.67663161	9.63776787	21.45169669	H
atom	1.17203072	8.59444117	21.46740122	H
atom	2.75565106	10.74776885	22.03496351	Cl

atom -0.56606359 12.67022635 18.09398803 Cl
atom -1.64293662 10.22260374 18.66012887 H
atom 2.79676906 5.27805929 18.12493911 Cl
atom 1.19396410 7.41510636 18.68427672 H
atom -1.65311512 5.75253046 21.44009191 H
atom -0.56793513 3.31482634 22.01385689 Cl
atom -0.53428887 2.74527279 18.08578954 Cl
atom 2.77173419 0.83457797 22.04463006 Cl
atom 3.28683654 1.08985574 18.11969156 Cl
atom 5.93990179 1.37555913 18.68989439 H
atom 5.95293812 4.67235427 21.44554963 H
atom 3.30058752 4.98833891 22.00667588 Cl
atom -0.77826182 5.65600603 20.81294523 C
atom -0.32907456 6.75239585 20.06114474 C
atom 0.84849920 6.60620408 19.31221353 C
atom 1.50858659 5.40540887 19.28423272 C
atom -0.05046980 4.49486808 20.84750170 C
atom -0.02937147 1.57282750 19.26473857 C
atom 1.50462850 0.68645777 20.86511471 C
atom 5.42543996 3.96805615 20.81818945 C
atom 6.14184330 3.02283000 20.06810642 C
atom 5.41818959 2.08434242 19.31717346 C
atom 4.04851568 2.13100378 19.28429923 C
atom 4.05557720 3.93696852 20.84716735 C
atom 1.11712016 4.28918367 20.06752663 C
atom 1.11986516 1.79081466 20.06494121 C
atom 3.28545481 3.03837534 20.06438389 C
atom 1.84282653 3.04241374 20.06578506 C
atom 15.62051349 0.27592032 18.65198809 H
atom 16.48368285 0.38334772 19.29334946 C
atom -7.46085782 13.57134722 21.47744865 H
atom -7.79873048 14.37274247 20.83589676 C

Optimized geometry for the PTM$_{acetylenic}$ 2D-CORF

lattice_vector 45.41400725 0.00000000 0.00000000
lattice_vector -22.80356264 39.49692908 0.00000000
lattice_vector 0.00000000 0.00000000 40.00000000
atom 3.13130275 11.24268833 22.01953761 Cl
atom 0.05009531 9.46119343 18.01043733 Cl
atom 3.15738688 1.74113315 18.01163695 Cl
atom 0.06127073 3.50519296 22.01698344 Cl
atom 8.27395549 8.28848181 18.01975671 Cl
atom 8.28050393 4.71273920 22.01406700 Cl
atom 22.75413488 9.46536433 18.01320678 Cl
atom 19.65986692 11.23324228 22.01825378 Cl
atom 22.75489901 3.50169195 22.01323961 Cl
atom 19.66916957 1.73494710 18.00108385 Cl
atom 14.53842718 8.27959309 18.01429778 Cl
atom 14.52991149 4.70036513 22.00551851 Cl

atom	4.61989151	8.54831919	22.12335199	Cl
atom	1.63219633	6.82024433	17.91749307	Cl
atom	4.63368978	4.44208146	17.91236748	Cl
atom	1.63178741	6.15305276	22.11507910	Cl
atom	5.19663917	8.22888692	17.92065406	Cl
atom	5.20309889	4.76578064	22.11714861	Cl
atom	21.18339472	6.81773538	17.91776337	Cl
atom	18.18362925	8.53222980	22.12063536	Cl
atom	21.17676789	6.14502426	22.11310040	Cl
atom	18.18480834	4.43163125	17.90442839	Cl
atom	17.61566700	8.21584257	17.91315284	Cl
atom	17.60737772	4.74897391	22.10646947	Cl
atom	0.37148322	12.48314214	20.00464404	C
atom	0.39804209	0.48725049	20.01717977	C
atom	0.97407720	11.43044902	20.00931394	C
atom	0.99633284	1.54240850	20.01626809	C
atom	1.67145775	10.21459963	20.01393676	C
atom	1.68913913	2.76093345	20.01537777	C
atom	2.73013220	9.99526277	20.90965426	C
atom	1.32958742	9.18532616	19.12230779	C
atom	2.74864183	2.98571701	19.12195321	C
atom	1.34148111	3.78769537	20.90762902	C
atom	3.41678676	8.79219895	20.91502353	C
atom	2.02517536	7.98732566	19.12176696	C
atom	3.42993338	4.19185098	19.11877146	C
atom	2.03183496	4.98873925	20.91048165	C
atom	3.08739258	7.75587893	20.01858448	C
atom	3.09418968	5.22598794	20.01536081	C
atom	3.82021218	6.49292664	20.01766768	C
atom	5.28057279	6.49660912	20.01824746	C
atom	6.01136945	7.30466463	19.12430830	C
atom	6.01440098	5.69125767	20.91214357	C
atom	7.39670350	7.31186192	19.12690087	C
atom	7.39971506	5.68710844	20.90775791	C
atom	8.11821857	6.49986546	20.01643799	C
atom	9.51997040	6.49996069	20.01439228	C
atom	10.73295017	6.49862191	20.01270934	C
atom	22.41332722	12.48795210	20.00346515	C
atom	22.43309915	0.48803286	20.00602794	C
atom	12.07842090	6.49631238	20.01144832	C
atom	21.81705959	11.43167470	20.00941257	C
atom	21.82969672	1.54027305	20.00669490	C
atom	13.29139878	6.49354424	20.01074934	C
atom	21.12620427	10.21205415	20.01387383	C
atom	21.13221464	2.75609350	20.00788260	C
atom	14.69315368	6.48997823	20.01021735	C
atom	21.47405087	9.18411685	19.12302721	C
atom	20.06774951	9.98777793	20.90862469	C
atom	21.47547425	3.78230593	20.90250783	C
atom	20.07289990	2.97903390	19.11380762	C
atom	15.41509293	7.30121223	19.12034093	C

atom	15.41123458	5.67489459	20.89975834	C
atom	20.78353453	7.98312514	19.12138750	C
atom	19.38641220	8.78169856	20.91310997	C
atom	20.78169855	4.98135471	20.90624664	C
atom	19.38806500	4.18314899	19.11163661	C
atom	16.80041212	7.29233713	19.11701658	C
atom	16.79657223	5.67732232	20.90336826	C
atom	19.72075209	7.74723249	20.01634357	C
atom	19.71998824	5.21706770	20.00990465	C
atom	17.53080368	6.48363846	20.01072636	C
atom	18.99119526	6.48229001	20.01252809	C
atom	3.05948889	37.57145189	22.01312853	Cl
atom	-0.01803959	35.80446921	17.99483548	Cl
atom	3.08596371	28.07941477	17.98268668	Cl
atom	-0.01516830	29.84015361	21.98558424	Cl
atom	8.21216379	34.61626359	18.01272434	Cl
atom	8.19972220	31.03125065	21.99873112	Cl
atom	22.68063152	35.80015245	18.01280052	Cl
atom	19.58782926	37.55490796	22.02474470	Cl
atom	22.69367096	29.83325437	21.99357425	Cl
atom	19.60737734	28.06866286	17.98091513	Cl
atom	14.46262433	34.60381518	18.01492030	Cl
atom	14.46360837	31.01403572	21.99673366	Cl
atom	4.54971308	34.87795576	22.10711584	Cl
atom	1.56615148	33.16491720	17.89226431	Cl
atom	4.56174317	30.78092786	17.88703655	Cl
atom	1.55495218	32.48780568	22.08801319	Cl
atom	5.13469788	34.56607875	17.90725687	Cl
atom	5.12247331	31.09329007	22.09564676	Cl
atom	21.10864527	33.15366662	17.90804193	Cl
atom	18.11059962	34.85410825	22.11782948	Cl
atom	21.10800815	32.47152922	22.10319331	Cl
atom	18.11584162	30.76178442	17.89311138	Cl
atom	17.53981845	34.54618349	17.91149715	Cl
atom	17.54121761	31.06830501	22.09556193	Cl
atom	0.29917776	38.81796699	20.00479249	C
atom	0.32778031	26.82179617	19.98844928	C
atom	0.90278965	37.76587070	20.00406681	C
atom	0.92432768	27.87793159	19.98698413	C
atom	1.60092424	36.55041429	20.00305396	C
atom	1.61573186	29.09725974	19.98597586	C
atom	2.65952043	36.32811570	20.89819450	C
atom	1.26062521	35.52480248	19.10665914	C
atom	2.67580182	29.32305710	19.09352043	C
atom	1.26647855	30.12357960	20.87812116	C
atom	3.34695547	35.12549928	20.89922302	C
atom	1.95705713	34.32727291	19.10175946	C
atom	3.35686660	30.52933534	19.09204489	C
atom	1.95671582	31.32468894	20.88281157	C
atom	3.01819618	34.09226884	19.99900472	C
atom	3.02060220	31.56241360	19.98965160	C

atom	3.74890389	32.82813373	19.99605466	C
atom	5.20926405	32.82818037	20.00034340	C
atom	5.94436868	33.63625129	19.10999242	C
atom	5.93887874	32.01876395	20.89406832	C
atom	7.32971978	33.63947444	19.11560981	C
atom	7.32420134	32.01063678	20.89270285	C
atom	8.04700556	32.82353664	20.00502517	C
atom	9.44878141	32.82067654	20.00682862	C
atom	10.66177130	32.81702762	20.00766982	C
atom	22.34510192	38.81369546	20.01785186	C
atom	22.37511259	26.82260308	19.98208250	C
atom	12.00726060	32.81360017	20.00787819	C
atom	21.74695717	37.75845361	20.01798499	C
atom	21.77005469	27.87391615	19.98467525	C
atom	13.22025264	32.81094847	20.00757615	C
atom	21.05410065	36.53994925	20.01713636	C
atom	21.07020487	29.08840707	19.98856992	C
atom	14.62202738	32.80925828	20.00613655	C
atom	21.40089228	35.51529863	19.12209640	C
atom	19.99529367	36.31312941	20.91082691	C
atom	21.41198102	30.11327922	20.88531149	C
atom	20.00930643	29.31098393	19.09626492	C
atom	15.34184758	33.62409733	19.11776718	C
atom	15.34230728	31.99315899	20.89293542	C
atom	20.70994038	34.31461082	19.11633719	C
atom	19.31354071	35.10722566	20.91122104	C
atom	20.71490237	31.31038294	20.89326632	C
atom	19.32131597	30.51333469	19.09813396	C
atom	16.72716180	33.61784151	19.11338327	C
atom	16.72766251	31.99820691	20.89550680	C
atom	19.64786680	34.07551316	20.01130377	C
atom	19.65159617	31.54576298	19.99873413	C
atom	17.45974632	32.80818326	20.00439895	C
atom	18.92012889	32.80949160	20.00498562	C
atom	0.00927136	22.63135213	17.99280908	Cl
atom	-3.09054304	24.39232668	21.99667832	Cl
atom	0.02314021	16.67158259	21.99670009	Cl
atom	-3.05616190	14.89182060	17.98541218	Cl
atom	-8.20265962	21.42642862	17.98858384	Cl
atom	-8.20672753	17.84960707	21.98202904	Cl
atom	-1.55697624	19.98095542	17.89556634	Cl
atom	-4.56252392	21.68903306	22.09678975	Cl
atom	-1.56105463	19.31131544	22.09198265	Cl
atom	-4.54633505	17.58528051	17.88355950	Cl
atom	-5.12564673	21.36751097	17.88840089	Cl
atom	-5.12902655	17.90293127	22.08364450	Cl
atom	-0.33363036	25.65019516	19.99022826	C
atom	-0.29678705	13.65078831	19.99986448	C
atom	-10.66050636	19.64488554	19.98491939	C
atom	-0.92989838	24.59390312	19.99184649	C
atom	-0.89942112	14.70346407	19.99563591	C

atom	-9.44752371	19.64093485	19.98680191 C
atom	-1.62043409	23.37408174	19.99346373 C
atom	-1.59743141	15.91895794	19.99226645 C
atom	-8.04574417	19.63896911	19.98640054 C
atom	-1.27079158	22.34735395	19.10195436 C
atom	-2.67960644	23.14796313	20.88692945 C
atom	-1.25644598	16.94757542	20.88498222 C
atom	-2.65610016	16.13847151	19.09655220 C
atom	-7.32490076	20.45058362	19.09596385 C
atom	-7.32653145	18.82527950	20.87632466 C
atom	-1.95924169	21.14520223	19.09947974 C
atom	-3.35899068	21.94075682	20.89047760 C
atom	-1.95292957	18.14507439	20.88654813 C
atom	-3.34352832	17.34108865	19.09210257 C
atom	-5.93959094	20.44383137	19.09300510 C
atom	-5.94116403	18.82979616	20.88027501 C
atom	-3.02174908	20.90694371	19.99409677 C
atom	-3.01495368	18.37685281	19.98951688 C
atom	-5.20803451	19.63717226	19.98769123 C
atom	-3.74762218	19.63981303	19.99064988 C
atom	25.81267281	24.41540030	21.98369107 Cl
atom	22.72686771	22.63976160	17.97550674 Cl
atom	25.82717184	14.91192128	17.98007046 Cl
atom	22.73449738	16.68464641	21.98423747 Cl
atom	30.95836061	21.44285146	17.98392084 Cl
atom	30.94604830	17.86988168	21.98073775 Cl
atom	27.30137849	21.72123095	22.08204230 Cl
atom	24.30937380	19.99893770	17.87697320 Cl
atom	27.30249673	17.61325345	17.87298824 Cl
atom	24.30399136	19.33321310	22.07450884 Cl
atom	27.88050703	21.39654246	17.88269471 Cl
atom	27.86921586	17.93592918	22.08124723 Cl
atom	23.04597509	25.65638302	19.98022136 C
atom	23.07462524	13.65958410	19.99635383 C
atom	23.65058678	24.60481106	19.97929983 C
atom	23.67056061	14.71604259	19.98987587 C
atom	24.34972543	23.38991241	19.97898029 C
atom	24.36140692	15.93565828	19.98362236 C
atom	25.41007893	23.16954167	20.87253896 C
atom	24.00753488	22.36228562	19.08559856 C
atom	25.41936981	16.15888740	19.08803863 C
atom	24.01371173	16.96480317	20.87312360 C
atom	26.09681595	21.96649831	20.87547155 C
atom	24.70325175	21.16434425	19.08254316 C
atom	26.10024354	17.36522294	19.08136348 C
atom	24.70365339	18.16612612	20.87245433 C
atom	25.76570736	20.93102971	19.97865131 C
atom	25.76585762	18.40099169	19.97657299 C
atom	26.49517616	19.66615631	19.97803273 C
atom	27.95557186	19.66449737	19.98057986 C
atom	28.69061007	20.46907115	19.08696710 C

atom	28.68515160	18.85672268	20.87577946	C
atom	30.07601173	20.47055100	19.09082276	C
atom	30.07041441	18.84694125	20.87274044	C
atom	30.79325938	19.65640199	19.98190479	C
atom	32.19502708	19.65190600	19.98353663	C
atom	33.40801315	19.64824648	19.98465798	C

Optimized geometry for the oxTAM$_{rs}$ 2D-CORF

lattice_vector	16.56216504	0.04390260	0.00000000	
lattice_vector	-8.32040424	14.32101634	0.00000000	
lattice_vector	0.00000000	0.00000000	40.00000000	
atom	9.82256627	0.39596149	19.46372531	C
atom	0.77672644	13.51496099	19.46465078	C
atom	-0.62671822	13.48029166	19.46443199	C
atom	7.00337122	0.34743796	19.46364689	C
atom	9.13222226	1.58408115	19.46275180	C
atom	7.00004036	12.32035724	19.46147401	C
atom	5.60174311	12.31665911	19.46211386	C
atom	7.69578005	13.53315337	19.46153252	C
atom	4.93005685	11.08390255	19.46276209	C
atom	4.92342834	13.54586330	19.46237162	C
atom	3.54927001	13.54220823	19.46376577	C
atom	2.87751787	12.30956559	19.46482210	C
atom	3.55589047	11.08039571	19.46430145	C
atom	6.97713109	9.90924159	19.46089165	C
atom	7.70239596	11.11129810	19.46111120	C
atom	9.10593838	11.14592987	19.46074863	C
atom	9.79637616	9.95783773	19.45927629	C
atom	7.66764230	8.72116905	19.45931711	C
atom	9.12572570	3.94414365	19.46112174	C
atom	7.72207134	3.97128374	19.46108464	C
atom	6.99020971	5.16935430	19.46096108	C
atom	7.67404679	6.36116901	19.46016589	C
atom	9.80951502	5.13595723	19.46075741	C
atom	11.85027492	6.32123992	19.45987055	C
atom	9.07120701	8.75576791	19.45841243	C
atom	9.07763721	6.33416459	19.45973749	C
atom	11.17198543	7.55035732	19.45859292	C
atom	9.77376161	7.54673589	19.45768872	C
atom	1.47921081	12.30609702	19.46550399	C
atom	7.72863603	1.54948468	19.46269419	C
atom	7.02629216	2.75860542	19.46185419	C
atom	-1.26772908	7.51750545	19.46127323	C
atom	-2.66607449	7.51386904	19.46064958	C
atom	-0.57205450	8.73028472	19.46244519	C
atom	-3.33773790	6.28110858	19.46000139	C
atom	-3.34447825	8.74301782	19.46075016	C
atom	-4.71862049	8.73933183	19.45922409	C
atom	0.83157697	8.70315586	19.46363365	C

```
atom     -1.30387374      9.92840873     19.46295562 C
atom     -0.62004157     11.12020742     19.46534908 C
atom      0.78364774     11.09318473     19.46588444 C
atom      1.51530116      9.89506067     19.46530103 C
atom     -1.29068871      5.10639496     19.46111811 C
atom     -0.56539324      6.30843229     19.46171837 C
atom      0.83819952      6.34309050     19.46265787 C
atom      1.52850426      5.15493951     19.46273664 C
atom     -0.60034489      3.91824675     19.46190800 C
atom     -0.59383475      1.55815896     19.46202411 C
atom      1.54157896      0.33289884     19.46282491 C
atom      4.94967543      3.98387023     19.46256528 C
atom      5.62800515      2.75464744     19.46272341 C
atom      4.95634767      1.52191517     19.46354289 C
atom      3.58217880      1.51837552     19.46351225 C
atom      3.57555558      3.98024215     19.46269711 C
atom      0.80317972      3.95288899     19.46266080 C
atom      0.80982326      1.53103514     19.46270581 C
atom      2.90386035      2.74752026     19.46305048 C
atom      1.50555447      2.74384468     19.46301024 C
atom     15.28441246      0.41027875     19.46176635 C
atom     -7.46269580     13.46210345     19.46187261 C
atom      1.52203220      7.52512200     19.46277569 O
atom      2.89407742      5.16365294     19.46314439 O
atom      5.62464606      5.17090378     19.46199525 O
atom      6.98369297      7.53930961     19.45838267 O
atom      5.61148656      9.90040182     19.46181667 O
atom      2.88084401      9.89347373     19.46589858 O
atom     -1.31049361     12.29836279     19.46606360 O
atom     -2.66947833      9.93001445     19.46131948 O
atom     -2.65623970      5.09773411     19.45886297 O
atom     -1.28423710      2.73632681     19.46207637 O
atom      2.90714647      0.33141119     19.46417854 O
atom      5.63777250      0.33853523     19.46388945 O
atom      9.81609054      2.76592424     19.46184203 O
atom     11.17512527      5.13431936     19.46066729 O
atom     -5.40022862      9.92268039     19.45839872 O
atom     -6.77234614     12.28396661     19.46159087 O
atom     11.18824434      0.40449543     19.46466973 O
atom     13.91883848      0.41190855     19.46129425 O
```

FHI-AIMS input file for single-point calculations (including basis set specification)

```
#############################################################################################
#
#  ground_states
#
#############################################################################################
#
#  Physical model
```

```
#
  xc                pbe0
#  spin              none
  spin              collinear
#  relativistic       none
  relativistic atomic_zora scalar
  charge            0
  default_initial_moment 0
  vdw_correction_hirshfeld
  k_grid            6 6 1
#
#  SCF convergence
#
  occupation_type    gaussian 0.01
  mixer              pulay
    n_max_pulay       10
    charge_mix_param   0.5
  sc_accuracy_rho    1E-3
  sc_accuracy_eev    1E-2
  sc_accuracy_etot   1E-5
  sc_accuracy_forces 1E-3
  sc_iter_limit      250
#
#  Relaxation
#
#  restart_relaxations .true.
#  relax_geometry   bfgs 1.e-2
#  RI_method        LVL_fast
#  relax_unit_cell   fixed_angles
# Output
#   output mulliken
   output hirshfeld_always
   output cube spin_density
#   output cube eigenstate_density  xx

# output DOS
  output dos -10. 5. 300 0.05
#dos_kgrid_factors 8 8 8

#   output band structure
  exx_band_structure_version 1
  output band   0.333333  -0.666666   0.000000   0.000000   0.000000   0.000000   100 K    G
  output band   0.000000   0.000000   0.000000  -0.333333  -0.333333   0.000000   100 G    k
  output band  -0.333333  -0.333333   0.000000   0.000000  -0.500000   0.000000   100 k    M
  output band   0.000000  -0.500000   0.000000   0.333333  -0.666666   0.000000   100 M    K


########################################################################################
#
#  FHI-aims code project
#  VB, Fritz-Haber Institut, 2009
```

```
#
#  Suggested "light" defaults for C atom (to be pasted into control.in file)
#  Be sure to double-check any results obtained with these settings for post-processing,
#  e.g., with the "tight" defaults and larger basis sets.
#
################################################################################
  species        C
#     global species definitions
    nucleus             6
    mass                12.0107
#
    l_hartree           4
#
    cut_pot             3.5  1.5  1.0
    basis_dep_cutoff    1e-4
#
    radial_base         34 5.0
    radial_multiplier   1
    angular_grids specified
      division   0.3326   50
      division   0.5710  110
      division   0.7727  194
      division   0.8772  302
#      division   0.9334  434
#      division   0.9625  590
#      division   0.9924  770
#      division   1.0230  974
#      division   1.4589 1202
#     outer_grid  974
     outer_grid 302
################################################################################
#
#  Definition of "minimal" basis
#
################################################################################
#     valence basis states
    valence      2  s   2.
    valence      2  p   2.
#     ion occupancy
    ion_occ      2  s   1.
    ion_occ      2  p   1.
################################################################################
#
#  Suggested additional basis functions. For production calculations,
#  uncomment them one after another (the most important basis functions are
#  listed first).
#
#  Constructed for dimers: 1.0 A, 1.25 A, 1.5 A, 2.0 A, 3.0 A
#
################################################################################
#  "First tier" - improvements: -1214.57 meV to -155.61 meV
```

```
     hydro 2 p 1.7
     hydro 3 d 6
     hydro 2 s 4.9
#  "Second tier" - improvements: -67.75 meV to -5.23 meV
#     hydro 4 f 9.8
#     hydro 3 p 5.2
#     hydro 3 s 4.3
#     hydro 5 g 14.4
#     hydro 3 d 6.2
#  "Third tier" - improvements: -2.43 meV to -0.60 meV
#     hydro 2 p 5.6
#     hydro 2 s 1.4
#     hydro 3 d 4.9
#     hydro 4 f 11.2
#  "Fourth tier" - improvements: -0.39 meV to -0.18 meV
#     hydro 2 p 2.1
#     hydro 5 g 16.4
#     hydro 4 d 13.2
#     hydro 3 s 13.6
#     hydro 4 f 17.6
#  Further basis functions - improvements: -0.08 meV and below
#     hydro 3 s 2
#     hydro 3 p 6
#     hydro 4 d 20
########################################################################################
#
#  FHI-aims code project
#  VB, Fritz-Haber Institut, 2009
#
#  Suggested "light" defaults for Cl atom (to be pasted into control.in file)
#  Be sure to double-check any results obtained with these settings for post-processing,
#  e.g., with the "tight" defaults and larger basis sets.
#
########################################################################################
  species        Cl
#     global species definitions
    nucleus             17
    mass                35.453
#
    l_hartree           4
#
    cut_pot             3.5          1.5  1.0
    basis_dep_cutoff    1e-4
#
    radial_base         45 5.0
    radial_multiplier   1
    angular_grids       specified
      division   0.4412  110
      division   0.5489  194
      division   0.6734  302
#      division   0.7794  434
```

```
#     division   0.9402  590
#     division   1.0779  770
#     division   1.1792  974
#     outer_grid  974
     outer_grid  302
################################################################################
#
#  Definition of "minimal" basis
#
################################################################################
#     valence basis states
    valence      3  s   2.
    valence      3  p   5.
#     ion occupancy
    ion_occ      3  s   1.
    ion_occ      3  p   4.
################################################################################
#
#  Suggested additional basis functions. For production calculations,
#  uncomment them one after another (the most important basis functions are
#  listed first).
#
#  Constructed for dimers: 1.65 A, 2.0 A, 2.5 A, 3.25 A, 4.0 A
#
################################################################################
#  "First tier" - improvements: -429.57 meV to -15.03 meV
     ionic 3 d auto
     hydro 2 p 1.9
#     hydro 4 f 7.4
     ionic 3 s auto
#     hydro 5 g 10.4
#  "Second tier" - improvements: -7.84 meV to -0.48 meV
#     hydro 3 d 3.3
#     hydro 5 f 9.8
#     hydro 1 s 0.75
#     hydro 5 g 11.2
#     hydro 4 p 10.4
#  "Third tier" - improvements: -1.00 meV to -0.12 meV
#     hydro 4 d 12.8
#     hydro 4 f 4.6
#     hydro 4 d 10.8
#     hydro 2 s 1.8
#     hydro 3 p 3
#  Further functions that fell out - improvements: -0.10 meV and below
#     hydro 5 f 14.4
#     hydro 4 s 12.8
#     hydro 3 d 11.6
#     hydro 4 s 4.1
################################################################################
#
#  FHI-aims code project
```

```
#  VB, Fritz-Haber Institut, 2009
#
#  Suggested "light" defaults for H atom (to be pasted into control.in file)
#  Be sure to double-check any results obtained with these settings for post-processing,
#  e.g., with the "tight" defaults and larger basis sets.
#
################################################################################
  species        H
#     global species definitions
    nucleus             1
    mass                1.00794
#
    l_hartree           4
#
    cut_pot             3.5  1.5  1.0
    basis_dep_cutoff    1e-4
#
    radial_base         24 5.0
    radial_multiplier   1
    angular_grids       specified
      division   0.2421   50
      division   0.3822  110
      division   0.4799  194
      division   0.5341  302
#      division   0.5626  434
#      division   0.5922  590
#      division   0.6542  770
#      division   0.6868 1202
#      outer_grid  770
      outer_grid  302
################################################################################
#
#  Definition of "minimal" basis
#
################################################################################
#     valence basis states
    valence      1  s   1.
#     ion occupancy
    ion_occ      1  s   0.5
################################################################################
#
#  Suggested additional basis functions. For production calculations,
#  uncomment them one after another (the most important basis functions are
#  listed first).
#
#  Basis constructed for dimers: 0.5 A, 0.7 A, 1.0 A, 1.5 A, 2.5 A
#
################################################################################
#  "First tier" - improvements: -1014.90 meV to -62.69 meV
     hydro 2 s 2.1
     hydro 2 p 3.5
```

```
#  "Second tier" - improvements: -12.89 meV to -1.83 meV
#     hydro 1 s 0.85
#     hydro 2 p 3.7
#     hydro 2 s 1.2
#     hydro 3 d 7
#  "Third tier" - improvements: -0.25 meV to -0.12 meV
#     hydro 4 f 11.2
#     hydro 3 p 4.8
#     hydro 4 d 9
#     hydro 3 s 3.2
########################################################################################
#
#  FHI-aims code project
#  VB, Fritz-Haber Institut, 2009
#
#  Suggested "light" defaults for O atom (to be pasted into control.in file)
#  Be sure to double-check any results obtained with these settings for post-processing,
#  e.g., with the "tight" defaults and larger basis sets.
#
########################################################################################
  species        O
#     global species definitions
    nucleus             8
    mass                15.9994
#
    l_hartree           4
#
    cut_pot             3.5  1.5  1.0
    basis_dep_cutoff    1e-4
#
    radial_base         36 5.0
    radial_multiplier   1
     angular_grids specified
      division   0.2659   50
      division   0.4451  110
      division   0.6052  194
      division   0.7543  302
#      division   0.8014  434
#      division   0.8507  590
#      division   0.8762  770
#      division   0.9023  974
#      division   1.2339 1202
#      outer_grid 974
      outer_grid 302
########################################################################################
#
#  Definition of "minimal" basis
#
########################################################################################
#     valence basis states
    valence      2  s   2.
```

```
   valence      2  p   4.
#    ion occupancy
   ion_occ      2  s   1.
   ion_occ      2  p   3.
################################################################################
#
#  Suggested additional basis functions. For production calculations,
#  uncomment them one after another (the most important basis functions are
#  listed first).
#
#  Constructed for dimers: 1.0 A, 1.208 A, 1.5 A, 2.0 A, 3.0 A
#
################################################################################
#  "First tier" - improvements: -699.05 meV to -159.38 meV
    hydro 2 p 1.8
    hydro 3 d 7.6
    hydro 3 s 6.4
#  "Second tier" - improvements: -49.91 meV to -5.39 meV
#    hydro 4 f 11.6
#    hydro 3 p 6.2
#    hydro 3 d 5.6
#    hydro 5 g 17.6
#    hydro 1 s 0.75
#  "Third tier" - improvements: -2.83 meV to -0.50 meV
#    ionic 2 p auto
#    hydro 4 f 10.8
#    hydro 4 d 4.7
#    hydro 2 s 6.8
#  "Fourth tier" - improvements: -0.40 meV to -0.12 meV
#    hydro 3 p 5
#    hydro 3 s 3.3
#    hydro 5 g 15.6
#    hydro 4 f 17.6
#    hydro 4 d 14
# Further basis functions - -0.08 meV and below
#    hydro 3 s 2.1
#    hydro 4 d 11.6
#    hydro 3 p 16
#    hydro 2 s 17.2
```

Bibliography

1. Speight, J. *Lange's Handbook of Chemistry, 70th Anniversary Edition*. (McGraw-Hill Education, 2005).
2. Li, Y. *et al.* Mapping the elastic properties of two-dimensional MoS2 via bimodal atomic force microscopy and finite element simulation. *npj Comput. Mater.* **4**, 49 (2018).
3. Fthenakis, Z. G. & Lathiotakis, N. N. Graphene allotropes under extreme uniaxial strain: An ab initio theoretical study. *Phys. Chem. Chem. Phys.* **17**, 16418–16427 (2015).
4. Lee, C., Wei, X., Kysar, J. W. & Hone, J. Measurement of the elastic properties and intrinsic strength

of monolayer graphene. *Science (80-.).* **321**, 385–388 (2008).

5. Alcón, I. & Bromley, S. T. Structural Control over Spin Localization in Triarylmethyls. *RSC Adv.* **5**, 98593–98599 (2015).

6. Gomberg, M. AN INSTANCE OF TRIVALENT CARBON: TRIPHENYLMETHYL. *J. Am. Chem. Soc.* **22**, 757–771 (1900).

7. Ballester, M. & Riera-Figueras, J. Inert Carbon Free Radicals. I. Perchlorodiphenylmethyl and Perchlorotriphenylmethyl Radical Series". *J. Am. Chem. Soc.* **4254**, 2215–2225 (1971).

8. Li, Z. *et al.* Cyclo-para-phenylmethine: An Analog of Benzene Showing Global Aromaticity and Open-shell Diradical Character. *J. Am. Chem. Soc.* **141**, 16266–16270 (2019).

9. Schnack, J. Molecular magnetism. in *Quantum Magnetism* (eds. Schollwöck, U., Richter, J., Farnell, D. J. J. & Bishop, R. F.) 155–194 (Springer, Berlin, Heidelberg, 2004). doi:10.1007/bfb0119593

10. Alcón, I., Reta, D., Moreira, I. de P. R. & Bromley, S. T. Design of multi-functional 2D open-shell organic networks with mechanically controllable properties. *Chem. Sci.* **8**, 1027–1039 (2017).

11. Santiago, R. *et al.* 2D Hexagonal Covalent Organic Radical Frameworks as Tunable Correlated Electron Systems. *Adv. Funct. Mater.* 2004584 (2020). doi:10.1002/adfm.202004584
